# IMplementing Predictive Analytics towards efficient COPD Treatments (IMPACT): protocol for a stepped-wedge cluster randomized impact study

**DOI:** 10.1186/s41512-023-00140-6

**Published:** 2023-02-14

**Authors:** Kristina D. Michaux, Rebecca K. Metcalfe, Paloma Burns, Annalijn I. Conklin, Alison M. Hoens, Daniel Smith, Laura Struik, Abdollah Safari, Don D. Sin, Mohsen Sadatsafavi, IMPACT Study Group, Nick Bansback, Prabjit Barn, Joan L. Bottorff, Stirling Bryan, Chris Carlsten, Mary De Vera, Andrea Gershon, Samir Gupta, Paul Gustafson, Mehrshad Mokhtaran, Jim Johnson, Phalgun Joshi, Janice Leung, Larry D. Lynd, Brian Simmers, Janet Sutherland, Dhingra Vinay

**Affiliations:** 1 Collaboration for Outcomes Research and Evaluation (CORE), Faculty of Pharmaceutical Sciences, University of British Columbia, Vancouver, British Columbia V6T 1Z3 Canada; 2 Centre for Health Evaluation and Outcome Sciences (CHÉOS), St. Paul’s Hospital, Vancouver, British Columbia Canada; 3 Centre for Heart Lung Innovation, University of British Columbia & St. Paul’s Hospital, Vancouver, British Columbia Canada; 4 Department of Physical Therapy, University of British Columbia, Vancouver, British Columbia Canada; 5 Patient partner, Vancouver, Canada; 6 School of Nursing, University of British Columbia, Kelowna, BC Canada; 7 Department of Mathematics, Statistics, and Computer Science, University of Tehran, Tehran, Iran; 8 Department of Medicine (Division of Respirology), University of British Columbia, Vancouver, British Columbia Canada; 9 Centre for Clinical Epidemiology and Evaluation, University of British Columbia, Vancouver, British Columbia Canada

**Keywords:** Protocol, Chronic obstructive pulmonary disease, Clinical prediction models, Decision aid, Process of care, Prescription appropriateness, Sex and gender

## Abstract

**Introduction:**

Personalized disease management informed by quantitative risk prediction has the potential to improve patient care and outcomes. The integration of risk prediction into clinical workflow should be informed by the experiences and preferences of stakeholders, and the impact of such integration should be evaluated in prospective comparative studies.

The objectives of the IMplementing Predictive Analytics towards efficient chronic obstructive pulmonary disease (COPD) treatments (IMPACT) study are to integrate an exacerbation risk prediction tool into routine care and to determine its impact on prescription appropriateness (primary outcome), medication adherence, quality of life, exacerbation rates, and sex and gender disparities in COPD care (secondary outcomes).

**Methods:**

IMPACT will be conducted in two phases. Phase 1 will include the systematic and user-centered development of two decision support tools: (1) a decision tool for pulmonologists called the ACCEPT decision intervention (ADI), which combines risk prediction from the previously developed Acute COPD Exacerbation Prediction Tool with treatment algorithms recommended by the Canadian Thoracic Society’s COPD pharmacotherapy guidelines, and (2) an information pamphlet for COPD patients (patient tool), tailored to their prescribed medication, clinical needs, and lung function. In phase 2, we will conduct a stepped-wedge cluster randomized controlled trial in two outpatient respiratory clinics to evaluate the impact of the decision support tools on quality of care and patient outcomes. Clusters will be practicing pulmonologists (*n* ≥ 24), who will progressively switch to the intervention over 18 months. At the end of the study, a qualitative process evaluation will be carried out to determine the barriers and enablers of uptake of the tools.

**Discussion:**

The IMPACT study coincides with a planned harmonization of electronic health record systems across tertiary care centers in British Columbia, Canada. The harmonization of these systems combined with IMPACT’s implementation-oriented design and partnership with stakeholders will facilitate integration of the tools into routine care, if the results of the proposed study reveal positive association with improvement in the process and outcomes of clinical care. The process evaluation at the end of the trial will inform subsequent design iterations before largescale implementation.

**Trial registration:**

NCT05309356.

**Supplementary Information:**

The online version contains supplementary material available at 10.1186/s41512-023-00140-6.

## Introduction

Chronic obstructive pulmonary disease (COPD), a progressive disorder of the airways, is one of the leading causes of mortality worldwide [1]. In 2012, COPD was responsible for an estimated three million deaths globally [1]. Over the disease course, COPD patients experience episodes of acute worsening of symptoms, called exacerbations or lung attacks [2]. These events, especially severe ones that require inpatient care, are major causes of morbidity and mortality [3, 4]. In Canada, severe exacerbations are one of the most common causes of medical hospitalizations, accounting for 93,353 hospital admissions in the 2017–2018 fiscal year [4]. Given the high health and economic burden, preventing exacerbations is a cornerstone of contemporary management of COPD and a major determinant of the choice of maintenance pharmacotherapy [5].

In current COPD management strategies and guidelines, such as those put forth by the Global Initiative for Chronic Obstructive Lung Disease (GOLD) [5] and the Canadian Thoracic Society (CTS) [6], treatment choice is based on a crude classification of the patient’s recent exacerbation history (e.g., whether the patient has been a “frequent exacerbator” in the previous 12 months). This approach, while intuitive and simplistic, has several drawbacks. First, it ignores other patient and disease characteristics that can improve the accuracy of exacerbation risk predictions, such as patients’ demographic characteristics, lung function, smoking history, and previous treatments [7]. Additionally, this method does not communicate a numerical estimate of risks, which can facilitate shared decision-making. It has been suggested that these limitations may be key reasons behind low adherence to guidelines among care providers, and low adherence to treatment among patients, as demonstrated in two recent studies in Canada [8, 9].

Precision medicine emphasizes the tailoring of disease management to patient characteristics and values to improve patient care and outcomes [10]. Clinical prediction models that objectively turn observed patient characteristics into actionable risk estimates are major enablers of precision medicine [11]. By quantifying the magnitude of risk and treatment benefit, clinical prediction models improve risk communication and informed decision-making between the patient and their care provider, potentially resulting in high satisfaction with, and adherence to, therapies [12]. However, an attractive feature of simple decision tools (e.g., frequent exacerbator definition) in the traditional patient-care provider encounter is that they can be applied without much added burden to the encounter. Historically, ease of use has been a component of clinical “sensibility” of clinical prediction models [13]. The increasing availability of electronic health records (EHR), which enable real-time data retrieval and automated risk calculation, should render the simplicity argument moot.

British Columbia (BC), a Canadian province with a publicly funded healthcare system serving a population of 5.2 million (as of 2021), is amid a major transition to a harmonized EHR platform. The *IM*plementing *P*redictive *A*nalytics towards efficient *C*OPD *T*reatments (IMPACT) study capitalizes on this window of opportunity to integrate a risk prediction model for COPD exacerbations into this EHR system for routine care and evaluate its impact on quality of care and patient outcomes. The risk prediction model of interest, named ACCEPT (*AC*ute *C*OPD *E*xacerbation *P*revention *T*ool) [14, 15], was designed specifically to address the shortcoming of previous studies and is considered the first COPD exacerbation clinical prediction model to be ready for clinical practice implementation [16].

### Objectives

The IMPACT study has two main objectives: (1) to integrate exacerbation risk prediction into routine COPD care by (a) designing a decision tool that incorporates ACCEPT and is embedded within the EHR and (b) developing an individualized informational handout for patients to address patient identified decisional needs, and (2) to determine the impact of the intervention on prescription appropriateness, medication adherence, quality of life, exacerbation rates, smoking cessation, and sex and gender disparities in COPD care.

## Methods

### Overall study design and setting

The IMPACT study will be implemented in two subsequent phases at two University of British Columbia (UBC) teaching respiratory clinics in Vancouver, BC, Canada: St. Paul’s Hospital and Vancouver General Hospital. During phase 1, we will conduct research with users to design an intervention that consists of a pulmonologist decision support tool that integrates the ACCEPT clinical prediction model into the EHR platform and an accompanying patient informational handout. When phase 1 is completed, we will conduct phase 2, a stepped-wedge randomized controlled trial (RCT) to evaluate the impact of the intervention on the process of care and on both patient-reported and clinical outcomes. The specific study design, population, and procedures for each of the two study phases are described separately in the following sections.

### Phase 1: Development of decision support tools for pulmonologist and patients

#### Study overview

**Fig. 1:**
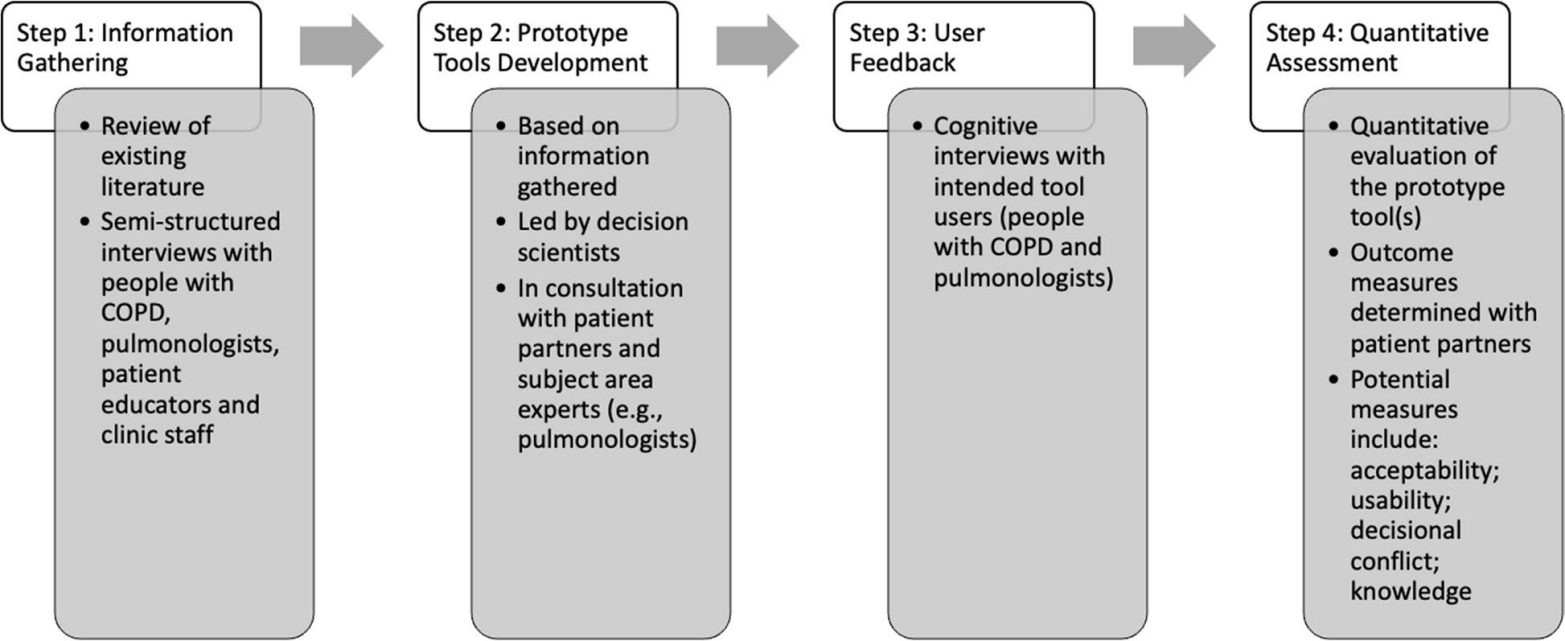
Steps in the decision support tool development for phase 1 of the IMPACT study. COPD, chronic obstructive pulmonary disease

Based on initial conversations with treating pulmonologists and patient partners, two tools will be developed to aid in the implementation of ACCEPT at point of care: (1) a decision support tool for pulmonologists, called the ACCEPT decision intervention (ADI), and (2) a printed informational handout for COPD patients that provides individual-specific advice given each patient’s predicted exacerbation risk and the prescribed medications (“patient tool”). Development of the ADI and the patient tool will follow the methods put forward by the International Patient Decision Aid Standards collaboration for systematic and user-centered development of decision aids [17, 18]. The development process is iterative, involves consultation with stakeholders (i.e., patients, pulmonologists, and clinic staff) throughout, and will be undertaken with integrated knowledge translation specialists in alignment with both the knowledge-to-action process model [18, 19] and the theory and frameworks comprised within the Theory and Techniques Tool (TTT) [20, 21]. Tool development will be divided into four related but distinct steps: (1) information gathering, (2) prototype building, (3) user feedback, and (4) pilot testing (Fig. 1).

#### Study population

Phase 1 study participants will fall into one of three groups: (1) pulmonologists, (2) individuals with COPD, and (3) clinic staff. Individuals with COPD will be eligible to participate if they have a diagnosis of COPD, are at least 18 years of age, and are able to speak and provide consent in English.

#### Tool development process

To ensure that the tools are relevant to the intended users, step 1 of tool development will identify the decisional needs and preferences of pulmonologists, and people with COPD, via semi-structured interviews. These decisional needs and preferences will inform the content and format of both tools. The interview guides will be developed collaboratively with subject area experts (e.g., decision scientists, specialists in smoking cessation) and intended tool users (e.g., pulmonologists, people with COPD). Interviews will be audio-recorded, and interview notes will be analyzed to identify preferred structure and content of the two tools. Specifically, at the end of each interview, the interviewer will compile a list of all content areas identified as important by the interviewee. For each tool, the list will be compiled across interviews with intended tool users. The complete list of all content areas identified will be discussed and reviewed with patient partners and pulmonologists on the study team to determine which content will be included in the study tools. Intended tool users will be recruited with the aim of maximizing variation in experience. For individuals with COPD, particular attention will be paid to diversity in age, sex, and gender, and time since diagnosis, based on feedback from our patient partners and the literature [22]. It is anticipated that between 4 and 6 pulmonologists will complete interviews, and between 10 and 20 individuals with COPD will be needed to reach saturation. Additional interviews will be conducted with clinic staff to assess the clinic workflow and assist in tool implementation.

Following the interviews, the initial prototype tools will be developed (step 2) using a collaborative process to draw on the unique skill sets and perspectives of different team members. At the center of this process will be decision scientists, integrated knowledge translation specialists, and COPD patient partners. The content and format of the tools will be derived from the semi-structured interviews and informed by the TTT [20, 21]. The TTT is an interactive resource that links specific behavior change techniques with mechanisms of action. It will be used to ensure that content is delivered in an evidence-based manner. Specific content will be developed with subject area experts on the study team (e.g., pulmonologists, smoking cessation experts) to ensure accuracy and alignment with best guidelines and practice principles. Patient partners and pulmonologists will provide input on the content and formatting before prototypes are developed, as well as provide feedback on prototypes to ensure the tools meet user needs and are easy to use and understand. Furthermore, following guidance from the International Patient Decision Aid Standards, the patient tool will target a grade 6 reading level [23].

The prototype tools will be revised and refined through cognitive interviews (step 3) [24]. Participants will be the intended tool users (i.e., pulmonologists will be interviewed for the ADI, and people with COPD will be interviewed for the patient tool). During the interviews, the relevant prototype tool will be presented to participants who will be instructed to navigate through the tool while stating their thoughts aloud. As needed during the interview, the interviewer will prompt the participant on specific aspects of the tool identified as important by the research team or previous participants. Interviews will be designed to elicit feedback on the layout, understandability of the language used, appropriateness of the content, and ease of use. When applicable, participants will be presented with previous versions of the relevant tools to assess the acceptability of potential changes based on previous participants’ suggestions. Cognitive interviews will continue until saturation is reached. For this work, saturation will be defined as no new significant concerns arising during cognitive interviews. It is anticipated that this will be reached after 5–10 interviews with each user group.

Following cognitive interviews, a preliminary evaluation of the patient tool (step 4) will be conducted. The metrics with which the tool will be evaluated will be determined in consultation with patient partners. Potential metrics include the following: knowledge, system usability [25], acceptability [26], and decisional conflict [27]. Quantitative evaluation of the ADI may be conducted after consultation with the study team. This evaluation would focus on usability and acceptability of the ADI. Results from quantitative evaluation will be shared with the study team, including intended tool users, to determine what changes, if any, are needed to the tool(s).

### Phase 2: A stepped-wedge cluster randomized control trial

#### Study design and setting

To test the effect of the intervention package (ADI and patient tool), we will conduct a prospective stepped-wedge cluster RCT. The unit of randomization will be staff pulmonologists at the two study sites. A stepped-wedge trial design was chosen for multiple reasons. First, clinical prediction model implementation studies should not randomize patients because of potential learning effects (changes in physician behavior after exposure to prediction model, even towards patients in the other group) [28]. Second, the stepped-wedge RCT is an implementation-oriented study design, since by the end of the trial all clusters will be assigned to the intervention arm.

**Fig. 2:**
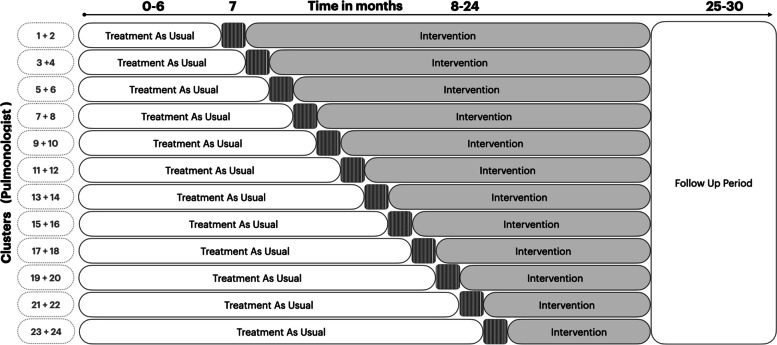
The stepped-wedge cluster randomized trial design of the IMPACT study. Notes: Patterned cells represent run in period (1 month)

In this trial, pulmonologists (clusters) will be assigned to treatment as usual (comparison arm) at the beginning of the study, with successive assignment to the intervention arm completed in a one-directional, staggered format. The one-way crossover from comparison to intervention will be allocated using a computerized random number generator and completed by the project manager during the first month of the study. Furthermore, due to the nature of the intervention, masking is not possible; thus, blinding will not be applied at the patient or cluster level. However, the allocation sequence will be concealed. Key study design features are shown in Fig. 2.

#### Study population

Since the intervention will be embedded into the EHR as a standard clinical decision support system and will be part of routine care, all staff pulmonologists at the two study sites (*n* = 28) will participate automatically in the study. However, conservatively anticipating data from the practice of four physicians will not be used for this study for various reasons, such as an extended leave; we aim to use data from 24 pulmonologists, who will comprise the study clusters. Individual patients who are referred to one of the participating pulmonologists at a study site with a physician-assigned diagnosis of COPD will be invited to participate in the clinical trial. For inclusion, individuals must be as follows: (1) a legal Canadian resident, (2) aged 18 years and older, and (3) able to understand English.

#### Intervention

**Fig. 3:**
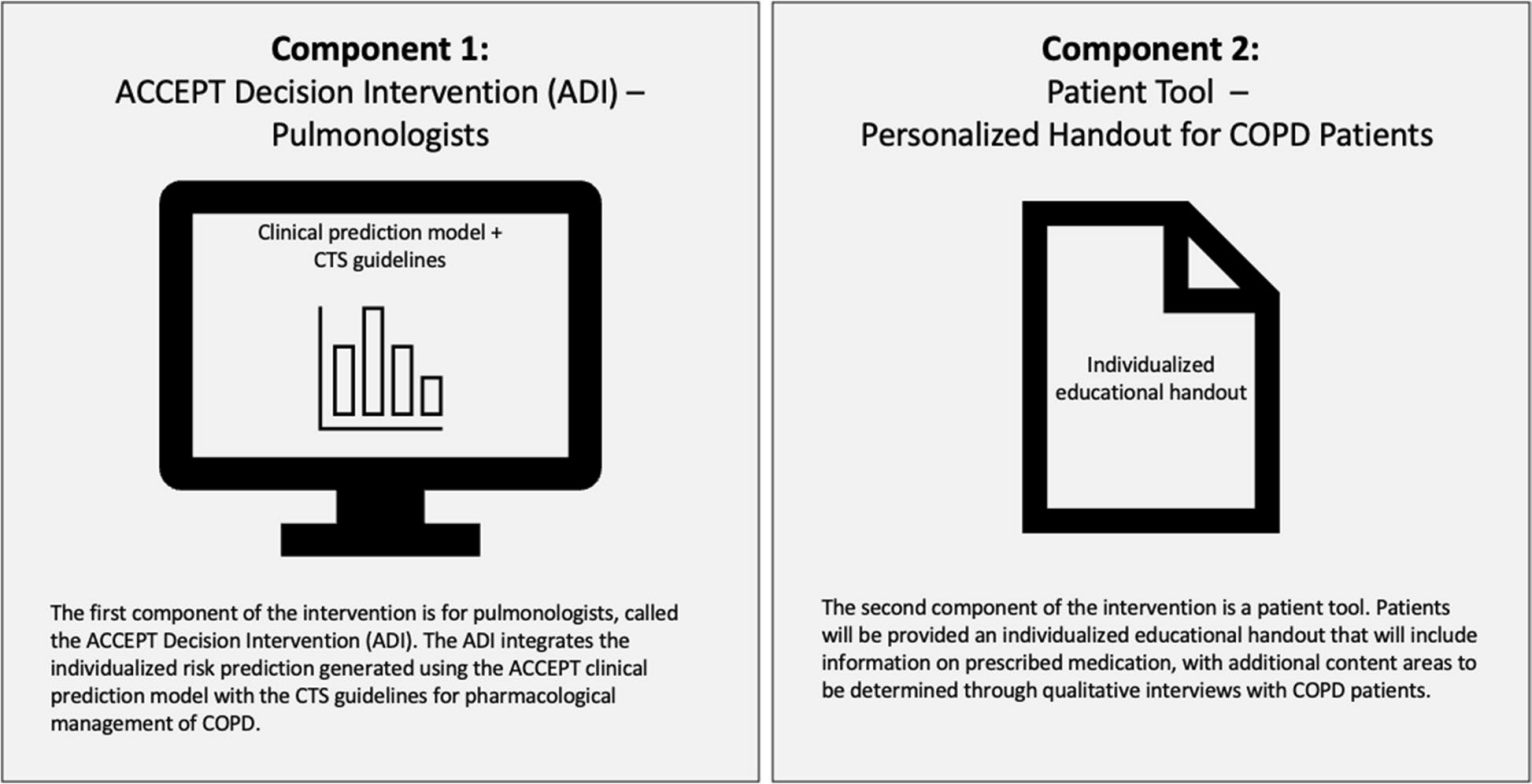
IMPACT study Intervention package. ACCEPT, Acute COPD Exacerbation Prevention Tool; ADI, ACCEPT decision intervention; COPD, chronic obstructive pulmonary disease; CTS, Canadian Thoracic Society

The study intervention has two related components that will be developed during phase 1: (1) the ADI, which is a software decision tool integrated within the EHR platform, and (2) a patient tool, which is a tailored educational handout for COPD patients (Fig. 3). The ADI combines risk prediction from ACCEPT with the CTS definition of low versus high risk for exacerbations. Features of the ACCEPT clinical prediction model, including details of the development and validation studies, are reported elsewhere [14]. In summary, ACCEPT uses routinely collected patient characteristics to predict the rate and severity of exacerbations in patients with COPD during the next 12 months. Predictors include sex, age, weight, height, smoking status, symptom burden, lung function, history of exacerbations, cardiovascular risk (represented by the use of statins), and current COPD therapies (some predictors are optional). In external validation, the latest version of ACCEPT (version 2.0, which will be implemented in this study) had a c-statistic of 0.73 (95% *CI*: 0.71, 0.76) for detecting the occurrence of any moderate and severe exacerbations. In comparison, exacerbation history alone had an area under the curve (AUC) of 0.67 (95% *CI*: 0.64, 0.70; *p* < 0.001) [15]. In decision curve analysis, ACCEPT provided positive net benefit over the use of exacerbation history alone over a large range of risk thresholds [15]. Several of ACCEPT’s predictor values will be prepopulated from the electronic patient chart, but pulmonologists will be given the option to override these values. To be aligned with the CTS guidelines, the frequent exacerbator group will be defined as those with predicted moderate exacerbation rate ≥ 2 per year or predicted severe exacerbation rate ≥ 1 per year.

The second component (the patient tool) will be a short, individualized patient information pamphlet. The information included in the pamphlet will be determined during phase 1 and will be tailored to each patient’s recommended treatment based on their ACCEPT score, their clinical needs, and their lung function.

#### Study outcomes

This is a complex trial, and we hypothesize that the overall benefits of the intervention will be multifaceted, including changes in the rate of under- and overtreatment, adherence to treatments, reduction in exacerbation rates, and narrowing of the known sex and gender gaps in COPD care [29, 30]. Table 1 describes the study’s primary endpoint and secondary outcomes and how each of these measures will help us to evaluate our hypotheses.

**Table 1:** Description of study outcomes and corresponding hypotheses evaluated by the clinical trial

Outcome label	Outcome	Outcome measure	Relevant hypothesis	Rationale for selection
Primary outcome (outcome 1)	Prescription appropriateness	The clinician prescription will be considered concordant if it is the same as the prescription based on the ACCEPT recommendation (or if ACCEPT suggests more than one eligible prescription, the clinician prescription is one of them); otherwise, it will be considered discordant	Reduction in under- and overtreatment	Reclassifying individuals from high to low risk will avoid expensive therapies in patients who will not benefit from them, while reclassification from low to high risk will result in more intense therapies that will reduce exacerbation risk
Outcome 2	Medication adherence	Medication adherence will be assessed in two ways: (a) using the medication possession ratio and (b) self-reported medication adherence. (a) Medication possession ratio is defined as the ratio of the total days’ supply dispensed to the total days’ supply prescribed during the study period. (b) Self-reported adherence will be assessed using the COPD-specific beliefs about Medicines Questionnaire (BMQ) [31]. The COPD BMQ is a 15-item scale with 2 subscales assessing patients’ perceptions of their need for medicine and concerns regarding medicine	Higher adherence of patients to treatment recommendations	We hypothesize that accurate risk communication will improve the adherence of patients to the recommended treatment [32, 33]
Outcome 3	Rate of moderate/severe exacerbations	Moderate exacerbations: Any outpatient physician visit for COPD followed by filling prescriptions for antibiotic or oral corticosteroids Severe exacerbations: A hospital admission with the main discharge code of COPD	Change in rate of moderate/severe exacerbations	The departure from guideline-base care is a mixture of over- and undertreatment, and the proposed intervention is likely to reduce such departure from guidelines. Correcting undertreatment will reduce exacerbation rate, while correcting overtreatment will not materially change the rate, resulting in a net-reducing effect. In addition, improving adherence will further reduce exacerbations
Outcome 5	Impact of COPD on health status	Measured by the COPD Assessment Test (CAT) [34]. The CAT includes 8 questions scored from 0 to 5, with higher scores indicating more severe impact of COPD	Improved health status	Patient-reported outcomes are likely to be affected by a quality-improvement intervention that involves addressing low health literacy and inaccurate medication beliefs [35]
Outcome 6	Quality of life	Euro Quality of Life-5 Dimensions (EQ5D). The EQ5D is a standardized questionnaires that assess 5 dimensions of health status, each at 3 distinct levels [36]. These data will be used to track changes in patient-report quality of life over the duration of the study	Improved quality of life	Patient-reported outcomes are likely to be affected by a quality-improvement intervention that involves addressing low health literacy and inaccurate medication beliefs [35]
Outcome 7	Smoking cessation	Measured by the following questions 1. Smoking status will be assessed by asking, “Do you currently smoke cigarettes” (yes/no)? 2. Motivation to quit will be assessed using the motivation to stop smoking (MTSS) scale [37]. Participants will be asked to classify themselves into one of the following six stages: (a) I do not want to quit smoking, (b) I think I should stop smoking but do not really want to, (c) I want to stop smoking but have not thought about when, (d) I REALLY want to stop smoking and intend to in the next 3 months; (e) I want to stop smoking and hope to soon, (f) I REALLY want to stop smoking and I hope to in the next month 3. Nicotine dependence will be assessed using the Fagerström test [38]—a questionnaire made up of 7 questions, the first scored as 0 and 1 (yes/no), and the remaining 6 multiple-choice questions scored from 0 to 3. The total test score ranges from 0 to 10, with higher scores indicating more intense physical dependence on nicotine 4. Cessation methods will be assessed by asking: Which of the following methods have you tried in the last 6 months (check all that apply): (a) nicotine replacement therapy, (b) cold turkey, (c) Champix®, (d) bupropion (Wellbutrin or Zyban), and or (e) behavioral support (e.g., smokers help line, cessation support group, cessation website, smartphone app)	Improved lifestyle behaviors	While smoking cessation is recommended for all current smokers with COPD, very few are offered such an intervention. Studies have suggested that even a brief intervention can be effective on reducing exacerbation rates [39]

*ACCEPT* Acute COPD Exacerbation Prevention Tool, *BMQ* Beliefs about Medicines Questionnaire, *CAT* COPD Assessment Test, *COPD* Chronic obstructive pulmonary disease, *EQ5D* EuroQoL 5-dimension, *MTTS* Motivation to Stop Smoking scale

We chose “prescription appropriateness” as the primary endpoint because it is the process of care outcome that is most directly affected by the intervention, and it has been frequently used in trials on computerized decision support tools [33]. Prescription appropriateness will be assessed by determining the concordance between the pulmonologist- and CTS-based treatment recommendations.

Secondary clinical outcomes include medication adherence (outcome 2) and rate of moderate or severe exacerbations (outcome 3). Data for the clinical outcomes will be gathered through questionnaires and chart reviews, as well as by linking patient data to BC’s administrative health databases using a unique personal health number; these databases comprehensively capture all healthcare encounters of all legal residents in BC at an individual level, independent of payer [40]. Medication adherence will be assessed using the medication possession ratio. For outcome 3, we will apply a validated definition of moderate and severe exacerbations; moderate exacerbations will be defined as any outpatient physician visit for COPD followed by filling prescriptions for antibiotic or oral corticosteroids, and severe exacerbations will be defined as any hospital admission with the main discharge code of COPD [41].

For patient-reported outcomes, we will assess self-reported medication adherence, quality of life, and smoking cessation using follow-up questionnaire data. Self-reported medication adherence will be assessed using a specific version of Beliefs about Medicines Questionnaire (BMQ COPD), which has been shown to have excellent construct validity and to be a strong predictor of adherence behavior [42]. For determining the impact of the intervention on the patients’ health status and quality of life, we will administer two questionnaires: (1) the COPD Assessment Test (CAT) and (2) the EuroQoL 5-dimension (EQ5D). The CAT assesses the impact of COPD symptoms on a person’s daily activities [34], and the EQ5D is a validated tool used to measure a patient’s overall quality of life [36]. Smoking cessation will be assessed by determining patients’ smoking status, motivation to quit [37], physical nicotine dependence [38], and methods used to quit in the last 6 months.

For the sex and gender effects, we will address two a priori hypotheses. First, we hypothesize that gender-related effects are, over and beyond biological sex, independent predictors of exacerbation risk, and their incorporation into risk stratification can improve predictive accuracy. Second, the extent of gaps in COPD care is known to be different between men and women [30], and we hypothesize that incorporating the decision tools into clinical care will change such sex/gender gaps. Our study includes novel data collection on gender identity, institutionalized gender, including socioeconomic status that will be assessed during the initial study visit (Supplementary Material — Sect. 1).

#### Study procedures

##### Recruitment, enrollment, and consent

At the beginning of the study (where all pulmonologists are assigned to the comparison arm), the project manager will review key information from the study protocol with participating pulmonologists. All pulmonologists will be emailed the latest CTS guidelines [6] and will be provided refresher training on the guidelines, if requested. Participation in these orientation interactions, however, will not be mandatory, and lack of participation by a pulmonologist will not exclude them from the study. In addition, before switching a given pulmonologist to the intervention arm, we will provide one-on-one training sessions on the use of the ADI and patient tool. For the ADI training, emphasis will be on how the software operates and how the ADI should be used and interpreted during clinical practice. For the patient tool, the training will include a detailed walk-through of the material, and pulmonologists will have the opportunity to customize the tool according to their practice preference, in a manner that does not affect the validity and consistency of the intervention (e.g., on which monitors they want to see the notifications, how large the fonts should be). A run-in period of 1 month is considered during which training and troubleshooting will take place (Fig. 2). Data gathered during this period will not contribute to the analysis.

**Fig. 4:**
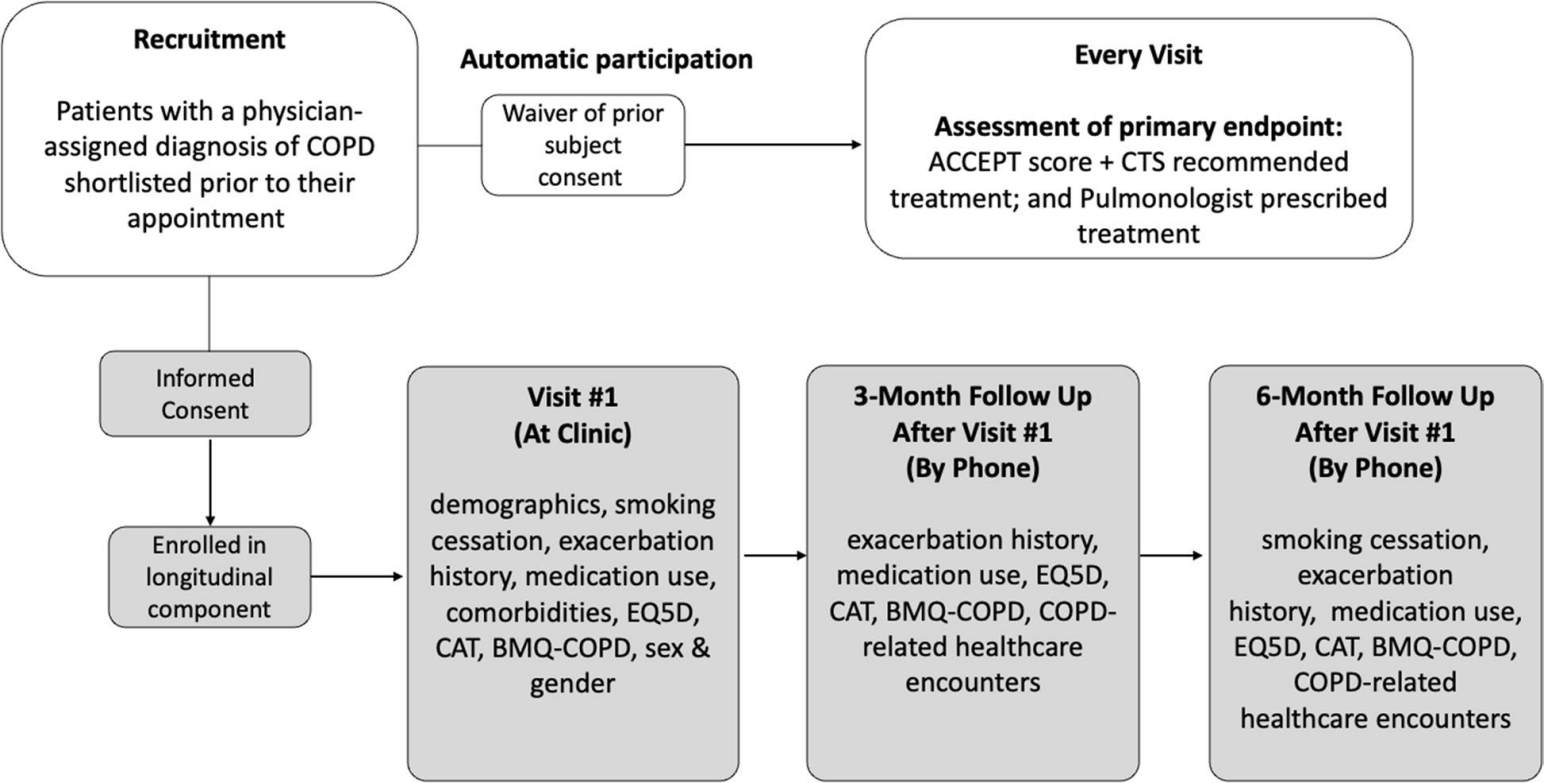
Participant flow chart. BMQ, Beliefs about Medicines Questionnaire; CAT, COPD Assessment Test; COPD, chronic obstructive pulmonary disease; CTS, Canadian Thoracic Society; EQ5D, EuroQoL 5-dimension

Data collected for the purpose of primary endpoint assessment will occur anonymously with a waiver of prior subject consent (Fig. 4). The alignment of prescribed medication with guidelines is directly targeted at measuring the relevant process-of-care variable. The information required to ascertain this endpoint is available through EHR and will be collected as aggregate data for analysis without recording any patient identifiers or changing any aspect of the COPD care being received by the individuals to whom the information relates.

Assessment of the secondary outcomes will involve recruitment of participants into a longitudinal component and linkage with administrative health data. An informed consent process will be applied here, with subjects simultaneously being given the option to opt out of the component of the study that involves accessing and linking their data to administrative health records. Patients who are eligible for the study will be shortlisted prior to their appointment based on their charts or referral notes by the research coordinator. At the end of each eligible appointment, the site coordinators will approach patients directly to invite them to participate in the longitudinal component of the study.

##### Data collection

The total duration of the trial is 30 months. Initially, all participating pulmonologists will be in the comparison arm. After 6 months, two randomly selected pulmonologists will be reassigned to the intervention arm every other month (Fig. 2). In month 18, the last two pulmonologists will be assigned to the intervention arm, and patient recruitment will continue until month 24. Follow-up data will be collected until month 30 to ensure 6 months of follow-up data for all patients.

To assess the primary endpoint, the prescribed versus recommended treatments will be automatically documented and collected in aggregate form under a unique identification (ID) code assigned to each pulmonologist. If a patient consents to the longitudinal component, a baseline study visit will be conducted that includes administration of questionnaires to collect clinical and patient-reported data to assess secondary outcomes (Fig. 4). Subsequently, two follow-up appointments will be arranged at 3- and 6-month post initial visit via telephone. If a patient sees an enrolled pulmonologist multiple times throughout the 24-month study period, the first visit with the pulmonologist will be used to mark the start of the follow-up period for longitudinal data collection, unless the pulmonologist was reassigned to the intervention arm between visits. In this case, if the patient has consented to the longitudinal component, the follow-up period will be reset during the first visit the pulmonologist is in the intervention arm, and the patient will be followed for another 6 months after the initial intervention arm visit.

To deliver the intervention package, site coordinators will set up automatic software triggers for all eligible patients seeing a pulmonologist allocated to the intervention arm. A trigger will launch when the pulmonologist opens a patient’s EHR chart during the patient encounter, and the ADI will be pre-populated with predictor values. If a predictor value is not retrieved from a patient’s EHR, the empty fields will be highlighted and will be fillable through dictation or by typing directly into the ADI computer interface. At the end of the encounter, the pulmonologist will print the customized patient tool that will be distributed by the research coordinator.

Regarding linkage with administrative health data, clinical data required for the assessment of secondary endpoints, including hospital visits, physician visits, medication prescriptions, and medication dispensing, will be requested in datasets from BC’s Ministry of Health. These data will be requested for 1 year before and 1 year after the baseline visit for each participant and will be used to assess medication adherence and frequency of exacerbations 12 months before and after study enrollment. These data will be received as anonymized and will be hosted by Population Data BC [40], who will provide remote access for data analysis and reporting.

##### Data monitoring and management

Before trial initiation, we will establish a Data Safety and Monitoring Committee (DSMC) to ensure that the rights and overall well-being of participants are safeguarded and attend to any immediate safety matters. Members of the DSMC will be selected by the study investigators but will act fully independently from the study, its investigators, and the funder. The DSMC will consist of individuals with methodological and clinical expertise.

Throughout the trial, study personnel will be available to troubleshoot and address questions about ADI use and triggers. Biweekly reports will be generated to track the frequency of ADI triggering, ADI completion, and proportion of recommendations overridden. This will allow us to determine the fidelity of ADI usage. If use of the ADI is lower than expected for a pulmonologist, we will approach them to determine potential issues.

All research data will be entered daily by the clinical research coordinators at each study site. We will use an electronic case report form for direct data entry into REDCap®. This minimizes data entry errors and improves data quality through the immediate application of data validation rules and boundary checking. Each record will be assigned a unique subject ID that will not include any personal or identifying information. A master list linking identifiable participants to research data will be kept encrypted and password protected under the secure storage system, and only the principal investigators will have access to the master list. Study statisticians and investigators will only have access to anonymized research data.

##### Process evaluation

The success of clinical decision support relies on an uptake of the tools into practice. To optimize uptake, it is critical to determine how the tools are being used by the patient-pulmonologist dyad. A sampled subgroup of patients (*n* = 20) and all pulmonologists (*n* = 24) will be invited for the qualitative process evaluation aiming to further improve risk communication and clinical workflow. Patients will be selected based their BMQ-COPD scores to ensure maximum variation in representation. After the patients complete a brief demographic questionnaire, we will conduct 1 h, semi-structured, telephone interviews to explore differences in patient experiences, assist with interpretation of trial findings, and provide direction for any further refinements to the intervention.

To gain insight into pulmonologist’s satisfaction, we will use normalization process theory (NPT) to better understand the factors that affected their implementation, with particular attention to clinical burnout and alert fatigue [43]. The 16-item NPT questionnaire covers questions in four important domains: sense-making, participation, action, and monitoring [43]. The information gained will be fed back to the design of the tools before largescale implementation.

#### Data analyses

##### Sample size calculations

Details of the sample size calculation is provided in the Supplementary Material — Sect. 2. In summary, the sample size is based on a formula for a stepped-wedged cluster RCT under a cross-sectional sampling method [44, 45], accounting for the variance in inflation due to the intra-cluster correlation and an adjustment for unequal cluster sizes. Sample size calculation was based on a type 1 error of 0.05 and a power of 80%. The current rate of prescription appropriateness was based on our analysis of BC’s administrative health data [46]. The unadjusted sample size was estimated to be 528 per arm.

The design effect is a function of the overall design features (i.e., the cross-over feature, number of clusters, run-in period), as well as the intra-cluster coefficients and cluster autocorrelation. These were obtained from a dedicated analysis of administrative health data from BC using prescription patterns from all physicians in the province [46]. The estimated intra-cluster coefficients and cluster autocorrelation were 0.026 and 0.491, respectively, resulting in a design effect of 2.15. Finally, the design effect was further adjusted for unequal cluster sizes [47], giving rise to a value of 2.18. Based on these, the total sample size is: 528 × 2.18 $\approx$ 1153.

To test the feasibility of achieving this sample, we performed an audit of the 2019–2020 fiscal year in one of the study sites and recorded 5559 visits. We estimate 17% of visits are due to COPD and eligible for this study. Given patient traffic in this site is expected to contribute to 60% of the overall traffic, and the 2-year recruitment window, the expected total number of eligible patients will be 3024. Therefore, to achieve the desired sample size, we will need to obtain the primary endpoint for 38% of the visits, a figure that we consider achievable given our experience with clinical studies at both sites.

##### Statistical analyses

Following an intention-to-treat principle, clusters will be analyzed according to their randomized crossover time regardless of whether crossover was achieved at the desired time. We will use a generalized linear mixed model (GLMM) in our analysis. This approach employs a random-effect term to account for multiple correlated visits for each pulmonologist (cluster), as well as other available covariates to adjust for potential confounders. Given the binary nature of the primary endpoint (prescription appropriateness), we will use a logistic GLMM. For our secondary outcomes, we will examine different standard continuous distributions in the GLMM to find the best fit to the data (according to the major goodness-of-fit measurement, such as Akaike information criteria [AIC], quasi-information criterion [QIC], and prediction error). The same approach will be used to find the optimal distribution and correlation structure of the GLMM analysis for each outcome. Additionally, time will be added as a fixed effect to capture trends in the outcome over the course of the study (steps) [44, 48]. Lastly, we will use multivariate imputation by chained Eqs. [49] to impute all missing values in the data that can be considered missing at random. All analyses will be adjusted for patient covariates to account for chance group imbalance. A sensitivity analysis will also be conducted that involves not adjusting for covariates (other than time of switch and cluster).

To test the sex- and gender-related hypotheses, we will conduct a principal component analysis in which gender-sensitive variables (Supplementary Material — Sect. 1) are combined into a scalar “gender index” [50]. To examine the effect of this index on the accuracy of risk prediction, we will use established statistical methods to examine the incremental value of a new predictor (improvement in c-statistic, bias-corrected net reclassification index, and decision curve analysis) [51].

For qualitative data analysis, interviews will be audio-recorded and transcribed, in addition to detailed note-taking throughout the interviews. The transcripts will be uploaded to NVivo 12 for analysis. The interviews will be analyzed using both inductive and deductive content analysis methods [52]. We will employ the TTT to guide the interviews, whereby questions are aimed at understanding barriers and facilitators to uptake and implementation of the tools. We will also employ the TTT to analyze the results of the interviews. For example, if a patient states that they feel they have improved skills to manage their COPD because of the instruction received, then we would deductively map this onto the appropriate TTT constructs. We will also inductively analyze interview data to capture contextual experiences described by the participants. We will disaggregate the data by sex (female/male) to accommodate possible differences in experiences. Two researchers will iteratively engage in the process of qualitative data analysis to ensure that a cohesive framework is achieved.

## Discussion

We reported on the protocol for the IMPACT study, which implements and evaluates an intervention that combines exacerbation risk prediction with current guidelines for the pharmacotherapy for the prevention of COPD exacerbation. The IMPACT study will be conducted in two phases. In phase 1, we will develop the intervention, which includes integration of exacerbation risk prediction into routine COPD care by designing a decision tool (ADI) that is embedded within the clinical workflow, and development of an individualized informational handout for patients (patient tool) to address patient-identified decisional needs. In phase 2, through a stepped-wedge cluster RCT, we will evaluate the effect of the intervention package on multiple outcomes, with the primary outcome being the alignment of prescribed medication with contemporary COPD guidelines. The unit of randomization will be practicing pulmonologists in the outpatient respiratory clinics of two teaching hospitals in Vancouver, BC. Secondary outcomes will include clinical outcomes (exacerbation rates and medication adherence) and patient-reported outcomes (COPD-related symptoms, general quality of life, and self-reported adherence). Separating enrollment for the data collection components of phase 2 will enable anonymized ascertainment of the primary endpoint while allowing subjects the option to participate specifically in the assessment of secondary outcomes. Linkage with the population-based administrative data will permit objective assessment of prescription filling and assessment of exacerbations during follow-up. A qualitative process evaluation component will assess enabling factors as well as barriers for uptake of the decision support tools. Through a formal stakeholder analysis in the formative stages of this study, we identified and engaged with three main knowledge user groups: health administrators in charge of the EHR systems, clinical guideline development committees (namely CTS), and patient groups (the Canadian Lung Association). In addition to peer-reviewed publications, we will directly communicate study findings and opportunities through these three main knowledge user groups, which have the capacity to influence clinical practice.

Clinical decision support systems have been shown to increase the proportion of patients receiving desired care by an average 5.8%, as shown by a systematic review of 122 controlled trials [33]. However, there is significant heterogeneity in these findings, with 25% of studies reporting improvements greater than 10%. While several study characteristics were associated with higher improvements, in a meta-regression, much of the between-study variability remained unexplained. We are optimistic that several aspects of our intervention increase its potential for improving care processes. One factor is a dedicated design and implementation phase with the involvement of providers and patients in the design of the decision support tools and changes in the clinical workflow [33]. Other features of our study that are associated with higher effects are low baseline rate of adherence to clinical guidelines [46], providing advice for patients (patient tool) in addition to practitioners and requiring practitioners to supply a reason for overriding advice [53]. Linking administrative health data to determine exacerbations and measure medication adherence in an objective way enhances the pragmatism element in the study design. Another important facet of this project is the explicit sex and gender lens added to the management of COPD. Despite a growing understanding that the burden of COPD is increasing in women, there is limited research on COPD and gender [54]. There is great scope to improve the care women receive for COPD, since they often do not receive treatments consistent with clinical guidelines [30].

Our study will also have limitations. First, as is typical with studies of clinical decision support tools [33], our trial is powered for the primary outcome (medication appropriateness), a process of care endpoint that is the immediate target of the intervention. As such, our trial is under-powered for detecting several secondary outcomes including a reduction in exacerbation rate. Second, while the risk of learning effect is generally low in our study (only one attending pulmonologist practices in the clinics in a given day, and ACCEPT is strictly available to the pulmonologist who is in the intervention arm [identified via system login]), some risk is inevitable. For example, the behavior of pulmonologists in the comparison arm might still be affected due to the awareness of a risk prediction tool for their colleagues. Furthermore, most outpatient care for COPD patients in Canada is offered by primary care physicians [55]. Finally, while we acknowledge that the biggest potential for impact lies in the community, we chose two hospital-based specialty outpatient clinics due to the recent implementation of harmonized EHR systems in the two settings. If we can demonstrate improvement in care processes and outcomes in an academic specialty care setting, there will be a strong motivation to expand this program to community care. Furthermore, quality of COPD care is not just decided by the choice of pharmacotherapy. Previous research on comprehensive patient-centred approaches in COPD management has generated encouraging results [55]. Despite this, these types of interventions have not been widely implemented due to significant resource requirements, including costs, and time required by personnel. Therefore, our choice of interventions and outcomes is based on feasibility (seamless integration into a busy clinic) and strong potential for impact. If successful, this project will create the necessary infrastructure to gradually expand the bundle of interventions.

Although there are many risk prediction tools for various health conditions and clinical outcomes, few of them have been tested in experimental studies. The design of clinical decision support tools that integrate risk prediction into routine care should arise from a collaborative, multidisciplinary approach which is understanding of clinician and team workflows and is informed by human factor engineering [56]. The absence of impact studies that demonstrate the utility of precision medicine tools is a major impediment to their uptake [57, 58]. The need for methodological and contextual expertise across several disciplines, requirement for coordinating numerous entities, and technological challenges are barriers against such studies. However, the recent confluence of several independent enabling factors, namely harmonization of EHR systems, suggests a unique window of opportunity to assess the viability and promise of EHR-enabled precision medicine in BC, with results and lessons learned potentially applicable to other jurisdictions.

### Study status

At the time of submission of this manuscript, recruitment and data collection for phase 1 are completed. The phase 2 clinical trial is expected to commence in January 2023. Protocol version 1.0. dated December 10, 2022, was used to prepare this manuscript.

## Supplementary Information

**Additional file 1: Section 1.** List of gender-sensitive variables. **Section 2.** Sample size calculation.

## Data Availability

All data collected as part of this study, except for the administrative health data, will be shared via the DRYAD initiative. The Dryad Digital Repository [59] is an online resource that makes data discoverable, freely reusable, and citable. We will share anonymized data that is free from patient identifiers or any sensitive data that might identify specific patients (e.g., extremely rare comorbidities). The linked administrative health data, however, will not be shared, as such data are under the stewardship of BC’s Ministry of Health and is protected by the provincial Freedom of Information and Protection of Privacy Act, which prohibits its sharing.

## Abbreviations

ACCEPT: ACute COPD Exacerbation Prevention Tool
ADI: ACCEPT decision intervention
AIC: Akaike information criteria
AUC: Area under the curve
BC: British Columbia
BMQ: Beliefs about Medicines Questionnaire
CAT: COPD Assessment Test
COPD: Chronic obstructive pulmonary disease
CTS: Canadian Thoracic Society
EHR: Electronic health records
EQ5D: EuroQoL 5-dimension
GOLD: Global Initiative for Chronic Obstructive Lung Disease
ID: Identification
IMPACT: *IM*Plementing *P*redictive *A*nalytics towards efficient *C*OPD *T*reatments
NPT: Normalization process theory
QIC: Quasi information criterion
RCT: Randomized controlled trial
TTT: Theory and Techniques Tool
