# Supplementary material for: IMplementing Predictive Analytics towards efficient COPD Treatments (IMPACT): protocol for a stepped-wedge cluster randomized impact study
Source: Diagn Progn Res. 2023 Feb 14;7:3. doi: 10.1186/s41512-023-00140-6 (PMC9926816; doi:10.1186/s41512-023-00140-6)
Supplement: Supplementary file 1 — Additional file 1: Section 1. List of gender-sensitive variables. Section 2. Sample size calculation. [file 41512_2023_140_MOESM1_ESM.docx]

**Supplementary Materials for “***IMplementing Predictive Analytics towards efficient COPD Treatments (IMPACT): Protocol for a stepped wedge cluster randomized impact study***”**

## List of gender sensitive variables

**Supplemental Table 1.** Gender-sensitive variables to be collected in the IMPACT clinical trial

| **Measure** | **Assessment / operationalization** | **Rationale for selection** |
| --- | --- | --- |
| **Gender** | | |
| Gender self-identity | 1. Open question: “what is your gender?” | Since a person’s gender self-identity is a fluid concept along a continuum, good practice of SGBA recommends using an open-ended question for gender.^1^ |
| Institutionalized gender | 1. Civil status (single; married or domestic partnership; divorced; separated; widowed) 2. Personal income 3. Level of education | Gender is also an institutionalized construct that reflects the socially ascribed statuses given to women and men based on biological sex. For women, marriage holds a high social value and often the only access to economic resources because traditional roles for women are to be homemakers, wives, and mothers. In the employment context, women receive less income than men for the same job position and qualifications. Canadian women in 2018 earned $0.87 for every dollar earned by men. These variables were used in the GENESIS-PRAXY study on CVD.^1^ |
| Gender roles | 1. Primary earner status (Are you the primary earner in your household?) 2. Employment status (Employed full-time; employed part-time; seeking opportunities, retired; self- employed; not working) 3. Primary caregiver (child, dependent) 4. Primary responsibility for housework 5. Job linked to exposure of VGDF | Social roles that are ascribed to women differ significantly from those ascribed to men based on binary assumptions of what is appropriate for human females and males. Traditional roles given to men include being responsible for full-time employment as the household ‘breadwinner’, whereas gender roles given to women include being the homemaker who either does not work, or works part-time and is responsible for being the primary caregiver and for domestic labor. Women are disproportionately more exposed to environmental toxins (VGDF) of cleaning products due to their higher burden of housework. These variables were used in the GENESIS-PRAXY study on CVD.^1^ |
| **Socioeconomic status (SES)** | | |
| Education | Categorical question:   1. Primary school 2. Middle school 3. High school (with diploma) 4. Some college/university (trade/professional/community) 5. Four-year college/university 6. Postgraduate degree (above bachelor) | Lower education is independently associated with acute COPD exacerbation, as well as greater disease severity, poorer lung function, and greater QoL issues.^2^ Education disparities between women and men exist in relation to obesity,^3,4^ which is a significant risk factor for COPD in women.^5^ |
| Occupation | 1. Open question: “what is your occupation group?” 2. Open question: “What is your job title? | Among people with COPD, occupational exposures and work disability attributed to lung disease, especially in combination, have been reported as risk factors for adverse health outcomes.^6^ Occupational exposures to VGDF products will differ by gender; manufacturing, construction or agricultural occupations are traditionally reserved for men as jobs in these industries are perceived as masculine appropriate. By contrast, other high-VGDF occupations such as cleaning services, textile work, or care-giving (e.g., nurses) are traditionally considered more feminine jobs where women are the predominant workers. These different occupational exposures are captured in the Occupation survey of the European Union Respiratory Health Survey II. |
| Income | Same categories as European Community Respiratory Health Survey II (highest category >150k) | Lower household income is independently related to COPD exacerbations and other adverse outcomes;^2^ this SES indicator was more strongly linked to Canadian men’s COPD outcomes.^5^ |
| Material circumstances | 1. In general, do you have enough money for your needs? 2. How often in the last 12 months, did it happen that you and other household members eat less because there is not enough food? 3. How much difficulty in the last 12 months do you/anyone in your household have/has to meet the payments of your bills (none, very little, slight. Some, great, very great) 4. In the past 12 months, was there a time you/anyone in your household needed to see a doctor or dentist, or fill a prescription, but did not do so because of cost? | The Materialistic Theory of SES and COPD was supported by a Swedish study finding that access to material resources was more relevant to COPD risk than low relative income.^7^ COPD in women has been shown to have cross-sectional and longitudinal associations with objective measures of obesity and weight gain, independent of conventional SES measures.^3,4^ These measures have policy relevance as they are included in the current Poverty Tracker used by the City of New York. Food security is another gender-specific determinant of COPD risk factors as correlations between food insecurity and weight status were also observed repeatedly in women only. |
| Asset ownership | 1. Do you own your own home? (yes, no-private rental, no-public housing, coop) 2. Do you own/lease a car? (y/n) | Previous research has documented the utility of home ownership as a measure of wealth in older populations, and wealth is considered a better measure of socio- economic status than income as a strong determinant of health. Older women often own fewer assets than older men due to discriminatory gender relationships and institutionalized gender. We have previously demonstrated that housing tenure was strongly associated with obesity in older adults, particularly in men.^3^ |
| Geographic location | Categorical question:   1. Large city 2. Suburb near large city 3. Small city or town 4. Rural area 5. Remote area | An established measure of SES is geographic location; urban versus rural differences are commonly reported in chronic diseases, and COPD specifically.^8^ There are distinct geographic regions in BC that are relevant to inequalities in access to healthcare and other resources for patients with COPD. |
| **Comorbidity** | | |
| Depression and anxiety | PH9 or CESD | Women with COPD demonstrate higher levels of depression and anxiety as well as worse symptom-related QoL; notably, QoL and disease symptoms are stronger determinants of COPD in women than physiologic or biologic measures.  In the Evaluation of COPD Longitudinally to Identify Predictive Surrogate Endpoints (ECLIPSE) cohort, cardiovascular comorbidity and diabetes mellitus were less prevalent in females, whereas osteoporosis, inflammatory bowel disease, reflux, and depression requiring treatment were more prevalent in females.^9^ |
| Cardiovascular disease |  |  |
| Osteoporosis |  |  |
|  |  |  |

CESD, Center for Epidemiological Studies Depression; COPD, Chronic Pulmonary Disease; CVD, Cardiovascular disease; PH9, Patient Health Questionnaire; QoL, Quality of Life; SES, Socioeconomic Status; SGBA, Sex and Gender-based Analysis; VGDF, vapors, gases, dusts, and fumes.

## Sample Size Calculation

## 2.1 Cluster sizes – Vancouver General Hospital (VGH)

We have 16 and 12 clusters (pulmonologists) at Vancouver General Hospital (VGH) and St. Paul’s Hospital (SPH), respectively. **Figure 1** shows the size of the clusters at VGH based on the audit of VGH clinics in 2019-20 fiscal year. We expect a similar cluster size distribution at SPH.

**Figure 1.** Number of visits by pulmonologists at VGH respiratory clinic in 2019


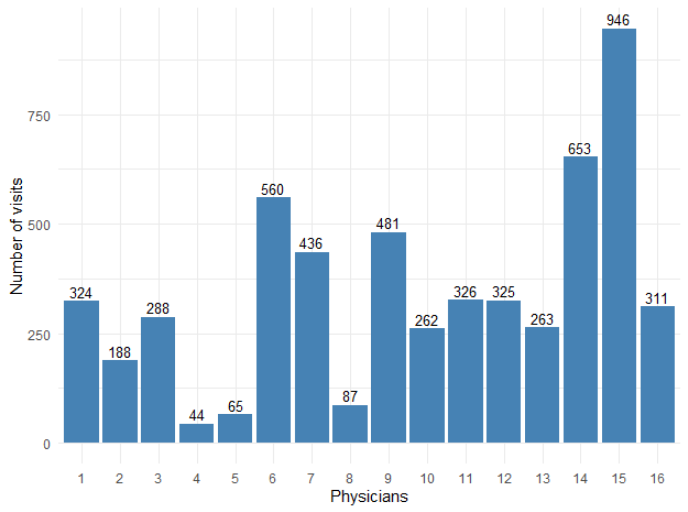


The total number of outpatient visits to the respiratory clinic at VGH during the above-mentioned 12-month period was 5,559. We conservatively assume that SPH patient traffic is 60% of VGH. An audit of 100 patient charts at SPH performed in February 2020 by Co-PI Don Sin demonstrated that 17% of patients visited the clinic for COPD. We take this proportion to be the same between the two clinics. These numbers will give an estimate of the COPD-related visits between the two clinics over two years as:

5559*1.6*0.17*2 = 3,024

## 2.2 Sample size formula

Based on the work by Hemming and Taljaard [HT]^10^ and Woertman et al,^11^ the sample size formula for a stepped-wedge cluster randomized clinical trial (SW-cRCT) under a cross-sectional sampling method is

$$N=N_{I}DE$$

where $N$ is the total required sample size, $N_{I}$ is the total sample size required under individual randomization, and $DE$ is the design effect, which can be obtained by the following formula:

$$DE=(t+1)\frac{1+\rho(ltm+bm-1)}{1+\rho(\frac{ltm}{2}+bm-1)}\frac{3(1-\rho)}{2l(t-\frac{1}{t})}$$

where $t$ is number of steps,$m$ is sample size per cluster per period, $\rho$ is intracluster correlation coefficient (ICC), $K=gt$ is total number of clusters, $g$ is number of clusters randomized per step, $M=m(t+1)$ is total sample size per cluster, $b$ is number of baseline measurements, and $l$ is number of measurements between each two consecutive steps.

The number of steps ($t$) needs to be fixed prior to the study. Depending on the experiment design and its constraints, we need to set either the total number of clusters, $K$, fixed (scenario B in HT), or the total sample size per cluster,$M$, (scenario A in HT). Given the nature of our study, the total number of clusters (i.e., the number of physicians) is fixed and known ($K=24$).

## 2.3 Parameters in our study

Given the design of our study, we will use the following values for the sample size formula:

$K=24$ : number of pulmonologists

$t=12$ : number of steps in which two randomly selected pulmonologists will be added to the intervention arm

$g=2$ : randomly selected pulmonologists will be added to the intervention arm at each step

$b=6$ : number of months before any pulmonologists will be added to the intervention arm

$l=1$ : number of months between every two consecutive steps

m = 4: by using the formula obtained from Appendix B of Hemming and Taljaard^10^

$N_{I}$ : total sample size required under individual randomization, which can be calculated for a given outcome. For example, for a binary outcome with $p_{control}=0.5$, $p_{exposure}=0.3$, $\alpha=0.05$, and $\beta=0.2$, the total estimated sample size based on comparing proportion of two samples is 188.

To estimate the required sample size for our study, we also need to estimate the ICC and the cluster auto-correlation (CAC). To estimate ICC and CAC, as described by Martin et al,^12^ we used a dedicated analysis of using prescription patterns from pulmonologists in BC.^13^ The cohort includes 6082 COPD patients with 23,403 pulmonologist visits, who were alive after 2010 and had at least one pulmonologist visit between January 2010 and March 2016. The estimated ICC and CAC were as follows:

$\rho=0.026$ : correlation coefficients between patients within each cluster (pulmonologist). The mathematical formula for this parameter is $\rho=\frac{S_{b}^{2}}{S_{b}^{2}+S_{w}^{2}}$, $S_{b}^{2}$ and $S_{w}^{2}$ are the variance between and within the clusters, respectively.

$CAC=0.491$ : the cluster auto-correlation. The mathematical formula for this parameter is $CAC=\frac{S_{b}^{2}}{S_{b}^{2}+S_{t}^{2}}$, where $S_{t}^{2}$ is the variance between patients within each time period and each cluster.

## 2.4 Sample size with unequal cluster sizes

To adjust the total sample size for a design with unequal cluster sizes, we need to multiply the estimated sample size for uniform cluster size by the ratio of the estimated variance of the effect sizes. In other words, we estimate the variance of the effect size for both scenarios (uniform and unequal cluster sizes), which is also known as relative efficiency (RE). We will then adjust our estimated sample size as follows:

$$SS_{Unequal}=SS_{Uniform}\times\frac{Var(\hat{\theta}_{Unequal})}{Var(\hat{\theta}_{Uniform})}$$

where $SS_{Unequal}$ is the estimated sample size with unequal cluster sizes, $SS_{Uniform}$ is the estimated sample size with uniform cluster size, $\hat{\theta}_{Unequal}$ is the estimated effect size with unequal cluster sizes, and $\hat{\theta}_{Uniform}$ is the estimated effect size with uniform cluster size. Equivalently, one can apply the RE to the estimated DE, and then use the adjusted DE to estimate the total sample size for a stepped wedge RCT design with unequal cluster sizes:

$${DE}_{Unequal}={DE}_{Uniform}\times\mathrm{RE}$$

To estimate the RE, we used the approximation given by Girling^14^ (equation 2, Section 3.1, and Section 4) as follows:

$$RE= \frac{1+\left( 1-CAC \right)*\bar{m}* \frac{ICC}{1-ICC}}{1+\left( 1+CV(m) \right)* \left( 1-CAC \right)*\bar{m}* \frac{ICC}{1-ICC}}\approx0.982$$

where $\bar{m}$ and CV(m) are the average and the coefficient of variation of cluster sizes, respectively. The adjusted DE (multiplied by the estimated RE) becomes 2.186, which approximately results in a total estimated sample size of 1153.

RCT sample size per arm (unadjusted): 527.339

RCT total sample size (unadjusted): 1056

Design effect (DE): 2.146

RCT sample size adjusted by DE: 527.339*2.146=1,132

Final RCT adjusted by both DE and unequal cluster sizes: 1153

## 2.5 Power as a function of participation rate

**Figure 2** presents the statistical power as a function of participation rate (proportion of expected eligible visits recruited in the trial). As figure 2 illustrates, given our study design setting described above, to achieve an 80% power, we should have a participation rate of 37.3%.

**Figure 2.** Statistical power versus participation rate for a stepped wedge cluster RCT.


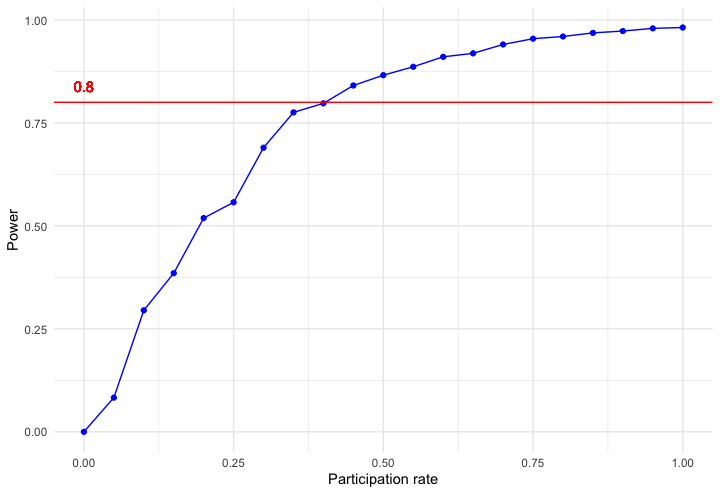


**References**

1. Pelletier, R. *et al.* Sex Versus Gender-Related Characteristics: Which Predicts Outcome After Acute Coronary Syndrome in the Young? *J. Am. Coll. Cardiol.* **67**, 127–135 (2016).

2. Eisner, M. D. *et al.* Socioeconomic status, race and COPD health outcomes. *Journal of Epidemiology & Community Health* **65**, 26–34 (2011).

3. Conklin, A. I. *et al.* Socioeconomic status, financial hardship and measured obesity in older adults: a cross-sectional study of the EPIC-Norfolk cohort. *BMC Public Health* **13**, 1039 (2013).

4. Conklin, A. I., Forouhi, N. G., Brunner, E. J. & Monsivais, P. Persistent financial hardship, 11‐year weight gain, and health behaviors in the W hitehall II study. *Obesity* **22**, 2606–2612 (2014).

5. Chen, Y., Breithaupt, K. & Muhajarine, N. Occurrence of chronic obstructive pulmonary disease among Canadians and sex-related risk factors. *Journal of Clinical Epidemiology* **53**, 755–761 (2000).

6. Blanc, P. D. The association between occupational factors and adverse health outcomes in chronic obstructive pulmonary disease. *Occupational and Environmental Medicine* **61**, 661–667 (2004).

7. Axelsson Fisk, S. & Merlo, J. Absolute rather than relative income is a better socioeconomic predictor of chronic obstructive pulmonary disease in Swedish adults. *Int J Equity Health* **16**, 70 (2017).

8. Croft, J. B. *et al.* Urban-Rural County and State Differences in Chronic Obstructive Pulmonary Disease — United States, 2015. *MMWR Morb. Mortal. Wkly. Rep.* **67**, 205–211 (2018).

9. Miller, J. *et al.* Comorbidity, systemic inflammation and outcomes in the ECLIPSE cohort. *Respiratory Medicine* **107**, 1376–1384 (2013).

10. Hemming, K. & Taljaard, M. Sample size calculations for stepped wedge and cluster randomised trials: a unified approach. *Journal of Clinical Epidemiology* **69**, 137–146 (2016).

11. Woertman, W. *et al.* Stepped wedge designs could reduce the required sample size in cluster randomized trials. *Journal of Clinical Epidemiology* **66**, 752–758 (2013).

12. Martin, J. *et al.* Intra-cluster and inter-period correlation coefficients for cross-sectional cluster randomised controlled trials for type-2 diabetes in UK primary care. *Trials* **17**, 402 (2016).

13. Bahremand, T. *et al.* Are COPD Prescription Patterns Aligned with Guidelines? Evidence from a Canadian Population-Based Study. *COPD* **Volume 16**, 751–759 (2021).

14. Girling, A. J. Relative efficiency of unequal cluster sizes in stepped wedge and other trial designs under longitudinal or cross-sectional sampling. *Statistics in Medicine* **37**, 4652–4664 (2018).
